# Development and Application of Performance Assessment Criteria for Next-Generation Sequencing-Based HIV Drug Resistance Assays

**DOI:** 10.3390/v12060627

**Published:** 2020-06-10

**Authors:** Michael G. Becker, Dun Liang, Breanna Cooper, Yan Le, Tracy Taylor, Emma R. Lee, Sutan Wu, Paul Sandstrom, Hezhao Ji

**Affiliations:** 1 National HIV and Retrovirology Laboratories, National Microbiology Laboratory at JC Wilt Infectious Diseases Research Center, Public Health Agency of Canada, Winnipeg, MB R3E 3R2, Canada; michael.becker@canada.ca (M.G.B.); Tracy.Taylor@canada.ca (T.T.); emmar.lee@canada.ca (E.R.L.); paul.sandstrom@canada.ca (P.S.); 2 ViroDx Clinical Diagnostics Laboratory, St. Louis, MO 63017, USA; liang@virodx.com (D.L.); Breanna.1.cooper@gmail.com (B.C.); lyan1211@gmail.com (Y.L.); 3 SutanStats, St. Louis, MO 63017, USA; titiwi@gmail.com; 4 Department of Medical Microbiology and Infectious Diseases, University of Manitoba, Winnipeg, MB R3E 0J9, Canada

**Keywords:** HIV, drug resistance, test, assay, next-generation sequencing, validation, assessment, criteria, benchmarks, standardization

## Abstract

Next-generation sequencing (NGS)-based HIV drug resistance (HIVDR) assays outperform conventional Sanger sequencing in scalability, sensitivity, and quantitative detection of minority resistance variants. Thus far, HIVDR assays have been applied primarily in research but rarely in clinical settings. One main obstacle is the lack of standardized validation and performance evaluation systems that allow regulatory agencies to benchmark and accredit new assays for clinical use. By revisiting the existing principles for molecular assay validation, here we propose a new validation and performance evaluation system that helps to both qualitatively and quantitatively assess the performance of an NGS-based HIVDR assay. To accomplish this, we constructed a 70-specimen proficiency test panel that includes plasmid mixtures at known ratios, viral RNA from infectious clones, and anonymized clinical specimens. We developed assessment criteria and benchmarks for NGS-based HIVDR assays and used these to assess data from five separate MiSeq runs performed in two experienced HIVDR laboratories. This proposed platform may help to pave the way for the standardization of NGS HIVDR assay validation and performance evaluation strategies for accreditation and quality assurance purposes in both research and clinical settings.

## 1. Introduction

HIV drug resistance (HIVDR) monitoring serves as a key component in effective HIV/AIDS management [1]. For improved antiretroviral therapy (ART) effectiveness, an HIVDR test is recommended to detect drug resistance mutations (DRMs) before ART initiation and after ART failure [2]. Conventional genotypic HIVDR assays rely on in-house or commercially available Sanger sequencing-based assays. Although Sanger sequencing has been widely applied as the “gold standard” for decades, there are some intrinsic limitations with this technology. Among its limitations are low data throughput and a poor ability to reliably detect minority resistance variants at frequencies below 20%. The clinical significance of low-frequency drug-resistant HIV-1 variants of <20% is still poorly defined but several key studies have shown an increased risk of antiretroviral treatment [3,4,5,6]. Next-generation sequencing (NGS) technologies are now broadly adopted in nearly all genetics-related fields, including HIV/AIDS genotyping. While sequencing chemistries vary, all NGS platforms share the commonality of high-output, clonal, and parallel sequencing. Not surprisingly, it has been well-documented that NGS-based HIVDR assays surpass the conventional Sanger approach in scalability, data throughput, and sensitivity for minority resistance variants [7]. Despite this, NGS-based HIVDR assays have been primarily limited to research laboratories and are rarely used in clinical settings, largely because the assay has not been standardized, which is necessary for accreditation by regulatory agencies. As such, only one complete NGS-based HIVDR testing platform, the Vela Sentosa^®^ SQ HIV-1 Genotyping Assay, has acquired regulatory approval for clinical use thus far.

Many evaluation strategies and criteria have been used for validation and performance assessment of molecular tests. For example, the World Health Organization (WHO) has developed a well-detailed validation strategy for Sanger-based HIVDR assays [8]. For more general applications, Burd [9] provides an in-depth summary of validation strategies and criteria that apply to molecular assays for infectious diseases. More recently, the Centers for Disease Control and Prevention also published a practical guideline for implementing NGS-based assays in a public health setting [10]. Although progress has been made in the field, an appropriate validation and performance assessment platform for NGS-based HIVDR testing has not been developed. There are multiple considerations when developing a validation platform for NGS-based HIVDR assays, including: (1) the significant genetic diversity of the HIV viral populations (or “quasispecies”) within an individual patient and between different patients; (2) the wide variety of library preparation protocols and bioinformatics pipelines used for NGS HIVDR testing; (3) the dual qualitative and quantitative nature of NGS HIVDR assays; and (4) the highly variable input viral loads and sample formats (serum, plasma, dried blood spot). 

By revisiting the relevant principles for molecular assay validation and considering the unique features of NGS-based HIVDR, here we propose a new practical analytical platform with clearly defined criteria and recommended benchmarks. Using a specifically constructed 70-specimen proficiency test panel, this proposed validation platform was used to assess the HIVDR assays performed by two experienced laboratories: ViroDx, a commercial Clinical Laboratory Improvement Amendments (CLIA)-certified Laboratory that routinely offers primary care clinical HIVDR testing; and the National HIV and Retrovirology Laboratories (NHRL) within the Public Health Agency of Canada, which is a WHO-accredited specialized HIVDR reference lab. The proposed platform can assess both the qualitative and quantitative features of NGS-based HIVDR assays, and could potentially be applied to validate such assays for clinical use.

## 2. Materials and Methods

### 2.1. Specimens

In the absence of an established proficiency test panel for NGS-based HIVDR testing, we developed a prototypic 70-specimen reference panel for this study. This panel was specifically constructed for the needs of NGS-based HIVDR assay validation and performance assessment, and it consists of plasmid mixtures at known ratios; well-characterized HIV isolates; infectious molecular clones; and REB-exempt, anonymized clinical specimens with known viral loads (Supplementary Table S1). We designed two plasmids with a 3500 bp insert from HXB2 coordinate 1901-5400. The wildtype plasmid is identical to HXB2 accession K03455, while the mutant plasmid contains 34 major drug resistance mutations: 13 in protease (PR) and 21 in reverse transcriptase (RT) (Supplementary Table S1). Plasmid concentrations were determined via qubit fluorometric quantification before serial dilution to obtain targeted DRM frequencies of 1%, 2%, 5%, 10%, and 20%. Replicates were generated with aliquots from the same mixtures. Our developed HIVDR reference panel meets the recommendations provided by Lee et al. [11].

### 2.2. NGS-Based HIVDR Assays

**Figure 1:**
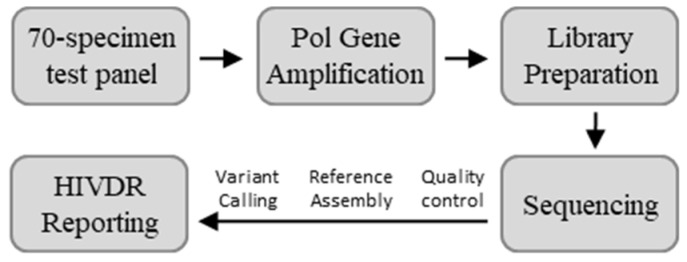
Workflow of the next generation sequencing (NGS)-based HIV drug resistance (HIVDR) testing platforms used in this study. Both participating laboratories started with the same 70-specimen test panel and used a similar approach, although the specifics varied.

The full panel was analyzed using Illumina MiSeq technology at both ViroDx (St. Louis, MO, USA) and the NHRL (Winnipeg, MB, Canada). Non-blinded sample processing for HIVDR testing was conducted in the two laboratories following their in-house-developed protocols, both following a similar workflow as outlined in Figure 1. 

Extractions of viral RNA were performed at NHRL as described in Taylor et al. [12]. In brief, samples were extracted in the NucliSENS^®^ easyMAG system (bioMérieux, Canada) as per the manufacturer’s instructions. An initial RT-PCR of the pol gene was performed using the SuperScript III one-step RT-PCR kit (ThermoFisher Scientific, Saint-Laurent, QC, Canada) with the following conditions: forward primer sequence 5′-GARAGACAGGCTAATTTTTTAGGGA-3′, reverse primer sequence 5′-ATCCCTGCATAAATCTGACTTGC-3′, and annealing at 53 °C. The pol gene was amplified from this PCR product and plasmids at NHRL using Phusion Hot Start II Hi-Fidelity DNA Polymerase (Thermo Fisher Scientific) with the following conditions for the nested PCR: forward primer sequence 5′-CTTTARCTTCCCTCARATCACTCT-3′, reverse primer sequence 5′-CTTCTGTATGTCATTGACAGTCC-3′, 35 PCR cycles, and a 10-minute final extension. Sequencing libraries were prepared using the Nextera™ XT DNA Library Prep Kit (Illumina, San Diego, CA, USA) and sequencing was subsequently performed on an Illumina MiSeq system using a V2 500-cycle kit. All the derived raw MiSeq data (in FASTQ format) were then processed using HyDRA, a commonly applied free pipeline for NGS-based HIVDR data analysis (https://hydra.canada.ca). The protocol used in the ViroDx lab was different from the NHRL method in the following: extractions of viral RNA were performed using the QIAamp viral RNA mini kit (Qiagen, Frederick, MD, USA) per the manufacturer’s instructions, amplification used a proprietary mix of primers, no nested PCR was performed, and a MiSeq V2 300-cycle kit was used for sequencing.

In total, the 70-specimen test panel was independently processed five times by experienced lab technicians. It was first processed in four separate experiments in the ViroDx lab by two independent technicians, followed by a single run at NHRL. 

### 2.3. Selection of Assessment Criteria for NGS-Based HIVDR Assays

We wanted a set of criteria to assess NGS-based HIVDR assays that are appropriate for the quantitative detection of HIV DRMs and that can address the high level of viral diversity observed in HIV infections. We considered the assessment criteria commonly used for Sanger-based HIVDR testing, along with accepted standards used to evaluate other NGS-based molecular tests [9,13,14,15,16,17,18,19,20]. A summary of the parameters that we considered suitable for the characterization and assessment of NGS HIVDR assays is provided below, which includes the DRM measuring interval, linear range, accuracy, sequence error rate, precision, analytical sensitivity, analytical specificity, limit of the viral load, and robustness.

DRM measuring interval: The DRM measuring interval is defined here as the range of HIV DRM frequency readouts that are reportable when considering errors due to non-linearity, imprecision, and sensitivity [18]. It is important to note the term measuring interval is often used interchangeably with reportable range [9,19,21]; however, we avoided the use of reportable range as it is used more commonly in molecular tests to describe its validated range of target genome coordinates [14,16]. 

Linear range: The linear range is often used for quantitative assays to determine the test range for which measured values are directly proportional to their expected values. Although linear range is sometimes used interchangeably with measuring interval and/or reportable range [9], that is only true for strictly quantitative tests and when the precision is still within an acceptable range [15,18,21]. 

Accuracy: Often used interchangeably with trueness [9,19], accuracy refers to the closeness of agreement between an observed test result and the expected or reference value. We intentionally avoided the use of the term trueness, as it has also been used previously to refer to systemic error (or bias) in molecular assays [15]. It should also be noted that for molecular tests, accuracy is sometimes used to refer to DNA sequence error rates [14,19]; however, we use the term sequence error rate (defined below) to refer to this quality metric. For HIVDR assays, we recommend that the term accuracy refers to the measurement of DRM frequencies.

Sequence error rate: We use the term sequence error rate to refer to compound errors in amino acid sequence reporting due to PCR errors, incorrect base calling, or artefacts introduced during data processing steps. We chose to use errors in the amino acid sequence rather than the DNA sequence since drug resistance is ultimately determined at the protein level. Amino acid-based HIV variant calling and analysis should be performed at the read level, as is considered best practice in NGS HIVDR assays [22]. To coincide with this, error rates should also be calculated using translated sequences. Additionally, using DNA sequence error rate as a measure underreports practical error rates, as an error within any of the three bases of a codon may affect its translation.

Precision: The extent to which repeated testing on identical specimens renders consistent results, both within the same run (repeatability) and between independent runs (reproducibility). Precision can further be subdivided into intra-run repeatability and inter-run, inter-operator, and inter-laboratory reproducibility [9,15,18]. We use precision to refer to the consistency of DRM frequency calls in NGS HIVDR tests.

Analytical sensitivity: For molecular tests, this refers to the probability that an assay will return a positive test result if a target sequence variant is present, measured as “1 − false-negative rate” [14,19]. For HIVDR assays, we recommend the term be used to refer to the probability of detecting a DRM when it is present. The reported value for analytical sensitivity should be the average value across all DRM variants tested.

Analytical specificity: For molecular tests, this refers to the probability that an assay will not detect target sequence variants if they are absent, measured as “1—false-positive rate” [14,19]. For HIVDR assays, we recommend the term be used to refer to the probability that when DRMs are absent, the test result is correctly reported as negative. In other words, the reported value for analytical specificity equals the proportion of runs that do not have false positives.

Limit of the viral load: The lowest viral load at which the assay can still effectively identify DRMs in a sample. This recommended limit of the viral load should allow an approved HIVDR assay to be compatible with the majority of samples received in a clinical setting.

Robustness: To be effective in a clinical setting, a HIVDR assay should meet the above criteria with clinical samples containing any main HIV subtype(s), which we define here as robustness. The main HIV subtypes circulating globally are summarized well by Bbosa et al. [23]. 

### 2.4. Assessment of NGS HIVDR Assays with Each of the Proposed Criteria

The lower boundary of the DRM measuring interval (limit of detection for DRMs) was determined with a probit regression analysis as outlined by the Clinical and Laboratory Standards Institute (CLSI) standards EP17 [24]. Probit analysis is commonly applied when investigating binomial response variables (e.g., detected vs. not detected) in response to a quantitative variable (e.g., DRM frequency). Linear regression can then be used to determine the 95% confidence interval. To perform this analysis, we used data generated from plasmid mixtures with targeted HIV DRM frequencies of 0%, 1%, 2%, 5%, 10%, 20%, and 100%, and averaged the results from all five replicates. We used a DRM frequency of 1% as our detection cut-off since this is the recommended or default reporting threshold of several popular HIVDR tools, such as HyDRA [12] and Stanford’s HIVdb-NGS (https://hivdb.stanford.edu/hivdb), and a 1% DRM frequency cut-off has also been used previously in other clinical studies [4,5,25]. To improve resolution of our dataset, ViroDx also used plasmid mixtures with DRMs at ultra-low frequencies (0.5%, 0.75%, 1%, 1.25%, 1.5%, 1.75%, and 2%) and a detection threshold of 0.25%. For all experiments, we used a probit value of 6.64 to indicate our limit of DRM detection as this represents the lower boundary of the 95% confidence interval [26].

The linear range of our NGS-based HIVDR assays was determined as outlined by CLSI standards EP06 [27] by plotting our measured DRM frequencies against their theoretical values for each of our plasmid mixtures. To better capture the linear range at the lower end of these DRM frequencies, we first performed a log10 transformation on data before linear regression analyses and calculation of an R^2^ value.

The accuracy of our NGS HIVDR assays was determined using data from plasmid mixtures with theoretical DRM frequencies of 1%, 2%, 5%, 10%, 20%, and 100%. This metric captured inaccuracy caused by PCR and sampling biases, along with PCR and sequencing errors. As a measure of inaccuracy, for each replicate, we determined the average deviation between our measured value and the expected frequency of 34 HIVDR variants, represented as a percentage (referred to as percent error). We also used these plasmid mixtures to determine the assay precision. The coefficient of variation (% CV, or standard deviation represented as a percentage [28]) was used as a measure of precision and was calculated for technical replicates (intra-run), independent runs (inter-run), individual operators (inter-operator), and across institutions (inter-laboratory). For all calculations other than intra-run precision, triplicates were averaged. Similarly, we calculated precision (% CV) using 40 DRMs that were identified across 12 clinical samples.

The sequence error rates of our HIVDR assays were determined by processing pure well-characterized plasmids and viral RNA propagated from infectious clones. We compared our measured amino acid frequencies to the known reference sequence. Any discrepancy between the reference and the reported phenotypes from the NGS reads at all loci was considered as an error. The sequence error rate was measured for each amino acid variant call by subtracting its observed frequency from the theoretical frequency of 100% (100% − the observed frequency), and these values were averaged to obtain an overall error rate. For interpretation, a 1% sequence error rate suggests amino acid frequencies were misreported by an average of 1% due to errors in PCR, sequencing, and data processing steps. Note, this does not capture errors caused by PCR biases or sampling biases, as the input for this analysis is a clonal population.

For the determination of analytical sensitivity, we used a 1% DRM frequency as our detection threshold for the same reasons as outlined in our probit analysis. Analytical sensitivity was calculated using 1 − false-negative rate. The false-negative rate was determined by processing plasmid mixtures of varying DRM frequencies (1%, 2%, 5%, 10%, 20%, and 100%) and monitoring for missed detection of 34 known DRMs. Analytical specificity was calculated as 1—false-positive rate. To determine the false-positive rate, we used our HIVDR assays to process plasmids with no known DRMs, as well as viral RNA from infectious clones, and interrogated the output data for any incorrectly identified DRMs at multiple detection cut-offs.

The limit of the viral load and the robustness of HIVDR assays are not NGS-specific but are instead applicable to all HIV genotyping assays. Because of this, these features were not extensively investigated here, and to do so would require a much more extensive reference panel.

## 3. Results

### 3.1. Determining the Lower Boundary of the DRM Measuring Interval

We wanted to investigate how our own NGS HIVDR assays performed according to our recommended assessment criteria. To determine the lower boundary of our DRM measuring interval, we performed a probit regression analysis to determine the HIV DRM frequencies that can be reliably detected at a cut-off of >1%. Using plasmid mixtures, we were able to calculate a probit score of 6.48 with a 1% input DRM frequency and 8.09 at all other frequencies tested (2%, 5%, 10%, 20%, 100%). Because most of the input mixtures tested were positive, we performed linear regression using only the 1% and 2% DRM inputs. This indicated that the 95% confidence interval for DRM detection on plasmids was ≈1.07% and that HIV DRMs could be reliably detected by NGS (*p* > 99%) if the input DRM frequency was above 2% (Supplementary Table S2; Supplementary Figure S1). This was similar to the lower boundary of the confidence interval at 1.34% obtained by ViroDx when probit was performed using an extended range of ultra-low DRM frequencies to increase the resolution with a detection threshold of 0.25% (Supplementary Figure S1).

### 3.2. Linear Range for the DRM Detection

**Figure 2:**
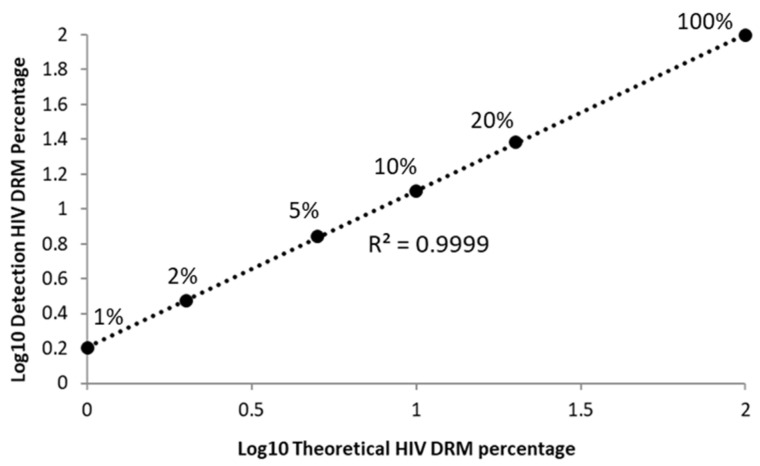
Linear range of detection for HIV drug resistance mutations (DRMs). The detected DRM percentage correlated well with input DRM frequency across our dataset range. Data show that HIVDR assays were biased toward overestimating the frequency of drug resistance mutations (likely due to an error in quantifying the input plasmids).

Next, we were interested in determining whether the HIV DRMs frequencies measured using our assays correlated with input HIV DRM frequencies. To accomplish this, we processed well-defined plasmid mixtures and plotted our measured DRM frequencies against their reference values (Figure 2). Across all frequencies tested, from 1–100%, input DRM frequencies were highly correlated with the values returned using our HIVDR assays (R^2^ > 0.999; *p*-value = 2.1 × 10^−9^ (ANOVA)), suggesting the quantification was reliable across our analytical range. 

### 3.3. Accuracy and Precision of the HIVDR Assays

**Figure 3:**
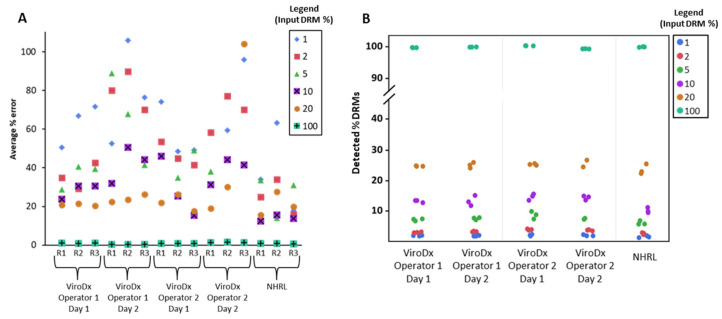
Accuracy and precision of HIVDR assays on plasmid mixtures with DRM frequencies of 1%, 2%, 5%, 20%, and 100%. The entire panel was performed twice by two independent operators at ViroDx, and once using the assay developed at the National HIV and Retroviral Laboratories (NHRL). Each HIVDR assay was performed in triplicate. (**A**) Inaccuracy as measured for each replicate (R1–3), operator, and for all mixtures. Each dot represents the average deviation/expected frequency × 100% (average % error) for that plasmid mixture. Overall, HIVDR assays are more accurate when measuring higher drug resistance mutation (DRM) frequencies, and the assay developed at NHRL had a lower overall error rate. (**B**) Observed DRM frequencies for all replicates, operators, and mixtures are color-coded by their expected value (see figure legend). Detected % DRMs refers to the average value across 36 individual DRM variants within each plasmid mixture and replicate. All assays overestimated DRM frequencies, suggesting systematic error due to plasmid mixing or assay performance. The intra- and inter-operator precisions were high, and are summarized further in Table 1.

We calculated the average percent error for all of our plasmid mixtures (Figure 3A) to gauge the accuracy of our HIVDR pipelines. Overall, assays were the most accurate at high DRM inputs and performed the worst at a 1% DRM input with a relative error ranging between 17.7–105.9%, with an average of 61.6%. At DRM inputs of 2%, 5%, 10%, and 20%, the average relative accuracy improved to 51.1%, 42.7%, 30.5%, and 27.8%, respectively. At all DRM frequencies, the accuracy was higher when using the NHRL’s HIVDR assay compared to the ViroDx assay, likely due to the differences in sample processing protocols. Across all replicates and for DRMs from 1–20%, the mutation frequency was overestimated when compared to their reference values (Figure 3B), which is suggestive of systematic bias.

The coefficient of variation was used to determine the intra-run, inter-run, inter-operator, and inter-lab precisions (Table 1). With an input of pure plasmid (100% DRMs), very little variability was observed (CV ≤ 0.3%). For all of the other DRM frequencies measured using plasmid mixtures, inter-lab variation was the most prominent factor affecting the precision, with CVs ranging from 7.7–15.9%. This was followed by intra-run precision with CVs from 6.2–12.7%, inter-run precision with 4.6–10.3%, and finally, inter-operator precision with 1.7–5.6%. This high level of intra-run variability was not surprising since both participating laboratories routinely perform HIVDR testing and the majority of the observed variation is likely derived from intra-run PCR and sampling biases. Similar patterns showing high intra-run diversity have been observed in the validations of other HIV-1 assays [29,30].

Additionally, we measured the precision of our assays using 12 patient samples across five independent runs to determine the level of precision that could be expected in a clinical setting (Supplementary Figure S2). When using clinical samples and analyzing DRMs with frequencies above 50%, the CVs were all below 16%, with the majority below 10%. At DRM frequencies below 50%, the CVs ranged from 4.2–48.5%, with an average of 26.8%; however, only a small number of DRMs were detected in this range (*n* = 7).

### 3.4. Assay Error, Analytical Specificity, and Analytical Sensitivity

We wanted to determine the overall assay error, which we defined here as the overall amino acid sequence error rate caused by PCR, sequencing, and/or data processing steps. To assess the assay error, we sequenced plasmids with a known sequence and measured the average frequency of erroneous amino acid calls. When measured using DNA plasmids, the assay error was low and ranged from 0.36% to 1.35%, with an average of 0.84% (Supplementary Figure S3). The results were similar when we processed viral RNA from infectious clones. Our assay error using viral RNA ranged from 0.51% to 1.41%, with an average of 0.89% (Supplementary Figure S3). This suggests that, regardless of input, our NGS HIVDR assays had a sequence error rate, at the amino acid level, of less than 1% on average due to errors introduced during sample processing and/or data processing steps. 

To determine the analytical sensitivity, we used a 1% DRM frequency as our detection threshold and monitored the detection of 34 known resistance mutations present in our plasmid mixtures at varying frequencies (Supplementary Table S1). At input DRM frequencies of 2% or higher, all 34 drug resistance mutations were detected in all runs and replicates, representing a sensitivity of >99% (Supplementary Table S3).

To measure analytical specificity, we performed our HIVDR assays on pure plasmid populations without any drug resistance mutations and monitored for the detection of DRMs, which indicates a false positive. With this approach, no false positives were detected (Supplementary Table S2), suggesting the analytical specificity was >99% and that detecting a specific DRM when it was absent is an infrequent occurrence at these detection cut-offs and using plasmids as the input. 

**Figure 4:**
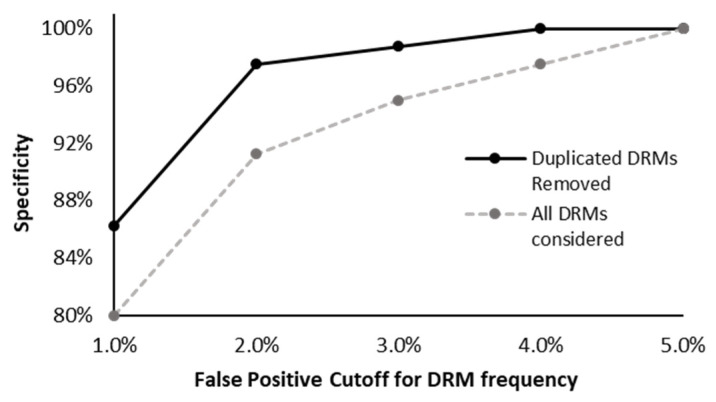
Assay specificity as determined using infectious clones with known drug resistance mutations. The specificity was determined using five different cutoffs to indicate the detection of a false positive drug resistance mutation (DRM): 1%, 2%, 3%, 4%, and 5%. If a run had one or more unexpected DRMs above this detection threshold, the entire run was considered to be a false positive (grey dashed line). As false positives can be introduced during the propagation of an infectious clone, we removed the DRMs observed across multiple independent replicates derived from the same infectious clone (solid black line). The majority of the observed false positives appeared to be the result of viral replication errors during the production of reference materials. At a 5% detection cut-off, specificity was 100% considering both the HIVDR assay and the propagation of the viral RNA.

We also measured the specificity using viral RNA derived from infectious clones, processing 15 different infectious clones in five independent runs. With this approach, we observed a false positive rate of 8.7% when considering DRMs with a measured frequency of 2% or greater, representing a specificity of 91.3% (Figure 4). While a lower detection threshold of 1% was applied, the specificity dropped markedly to 80%. Many of these false positives were observed across replicates, operators, and institutions (example in Supplemental Table S3), suggesting they may have been introduced before the NGS HIVDR assay and are likely the result of errors introduced during viral propagation. When these false-positive DRMs that were detected at very low frequencies but replicable across multiple runs were removed from our analysis, the specificity increased to 97.5% (2% DRM frequency detection cut-off) and 86.25% (1% frequency detection cut-off). A specificity of 100% was always observed when a detection threshold of 5% or greater was used (Figure 4).

## 4. Discussion

NGS-based HIVDR assays have been shown to outperform their Sanger counterparts on numerous metrics, including sensitivity; however, their transition from a research tool to a clinical test has been slow. There are many reasons for this, which have been summarized well by Ji et al. [31]. Multiple studies have done excellent work evaluating NGS-based HIVDR pipelines, including previous multi-laboratory collaborations [32,33]. However, these studies focus on the limits of DRM detection and do not provide concrete benchmarks for HIVDR assay standardization. None of the previous studies provided a holistic view of the validation methodology/criteria that best accommodate the unique features of the NGS HIVDR assays. To make the transition to NGS HIVDR a reality, we wanted to establish stringent performance assessment criteria to facilitate the characterization and performance assessment of NGS-based HIVDR assays. To be applied in a clinical setting, the assessment platform needs to consider the following: the high diversity of viruses like HIV, the qualitative and quantitative nature of the assay, and the reliability of output data such that it can be used to guide treatment in a clinical setting. Additionally, the assessment needs to be broad enough to accommodate the wide variety of independently developed NGS HIVDR test protocols that may vary significantly regarding sample inputs, nucleic acid processing steps, sequencing platform, and bioinformatics pipelines. 

To help formulate and validate an assessment platform for NGS-based HIVDR assays, we constructed a 70-specimen test panel and processed it with the assays established by the ViroDx and NHRL laboratories. Both the ViroDx and NHRL protocols have previously been shown to have a broad subtype coverage [12], which was not tested in this dataset. Using the data from our experimental runs, guidelines from the literature, and conclusions from the 2019 International Symposium on External Quality Assurance Strategies for NGS-based HIVDR testing, we propose criteria that could be used to validate and assess the performance of NGS HIVDR assays, along with recommended benchmarks values. These are described in more detail below and summarized in Table 2. We believe that our assessment criteria are broad enough that they can be widely applied for any subtype or combination of subtypes, and can address the diversity of HIV populations. 

DRM Measuring interval: We recommend that a HIVDR assay should reliably detect DRMs at frequencies within a measuring interval of 2–100%. As NGS-based HIVDR tests are highly sensitive, reporting at frequencies of 1% or lower is possible. However, we cannot recommend that DRMs detected below 2% are considered in a clinical setting for several reasons, including the overall assay sequence error rate of ≈1%, increased incidence of false-positive DRMs at frequencies below 2%, the limit of detection between 1–2% as determined using probit analysis, and the increased likelihood of cross-contamination and sampling/PCR biases. Our suggested lower limit of 2% coincides with recommendations provided during a recent comparison of HIVDR bioinformatics pipelines [34]. It should also be noted that the measuring interval may be affected by low input volumes or viral titers (to be discussed further), and would need to be adjusted accordingly. 

Linear range: The outputs from an NGS HIVDR assay should follow a linear range within the entire measuring interval, which we recommend should be 2–100%.

Precision: We recommend that the intra-run and inter-run imprecision of NGS-based HIVDR assays should be CV < 25% for DRMs at frequencies below 50%, and CV < 15% for frequencies of 50% or greater. We observed a marked decline in precision toward the lower end of our measuring interval, which is commonly seen in molecular assays [9]. Because of this, we chose benchmarks that we believe account for the expected variation when detecting low-frequency DRMs without compromising the integrity of the clinical test result. We also observed high intra-run imprecision, suggesting that the precision was largely affected by the PCR and sampling bias, or the PCR and sequencing errors. To address this, reverse transcription and PCR reactions can be performed in triplicate and combined before library preparation, which is similar to the common practice used in 16S amplicon-seq to increase precision [35]. Additionally, we recommend that assays have fully automated data processing steps to minimize the subjectiveness of NGS data management and the subsequent HIVDR reporting to maintain the precision between runs and operators. 

Accuracy: We recommend that the average percent error across the measuring interval should be below 40%, which is the level where we believe the inaccuracy could begin to affect the clinical interpretation. Note that this measurement is relative to the DRM frequency being measured and is not absolute, meaning that when measuring a true DRM frequency of 2%, its reported value should fall within 1.2–2.8%, and when measuring a true DRM frequency of 50% its reported value should fall within 30–70%. 

Sequence error rate: The overall error rate in the amino acid sequence derived from an NGS HIVDR assay should fall below 2%. If it is above this benchmark, the assay error can be minimized by using a high-fidelity reverse transcriptase and polymerase, switching to a more accurate sequencing platform, decreasing the number of cycles used for sequencing, or by applying more stringent sequence quality control strategies. 

Analytical sensitivity: At all DRM frequencies within the measuring interval, the sensitivity of the assay should be >99% for DRMs at frequencies of 2% or higher using a recommended detection threshold of 1%. We did not select a detection threshold of 2%, which represented the lower limit of our measuring interval, because it poorly reflected the sensitivity of the HIVDR testing. If we were to measure DRMs at a real frequency of 2%, also using a detection threshold of 2%, the highest sensitivity that could reasonably be achieved is 50% since half of the measured values would be expected to fall slightly below 2% and half slightly above. It should also be noted that the sensitivity was determined exclusively using plasmid mixtures, which produced more favorable results than could be expected using clinical samples consisting of viral RNA of variable quality. Because of this, our recommended benchmark refers exclusively to tests using plasmid mixtures and will need to be revisited once more extensive reference materials are available.

Analytical specificity: At all DRM frequencies within the measuring interval, the specificity of the assay should be >95% using a recommended detection threshold of 2%.

Limit of the viral load: In line with the current recommendations from WHO for existing Sanger-based HIVDR assays [8,36], NGS-based HIVDR assays should be capable of detecting viral loads of 1000 copies/mL or greater. This allows the HIVDR assay to perform in most cases of untreated HIV infection or ART failure [37,38]. However, precaution needs to be taken when viral loads are low since the accuracy of HIVDR assays is affected by sampling biases (to be discussed further). This makes the accurate detection of minority resistance variants challenging at low inputs.

Robustness (qualitative): Many factors influence the robustness of a molecular test, including the input sample types and quality, and overall operating procedures (e.g., amplification efficiency). Many of these factors will likely be reflected in the precision, accuracy, and DRM measuring interval. However, at the very least, we recommend that a validated protocol should be compatible (meet the above requirements) with any major HIV subtype(s) to be applied in a clinical setting. This assessment criterion is essential since HIVDR assays must have primer target sites that can manage the diversity of HIV in a single patient or between patients. 

We believe we have established criteria that will help to ensure that an NGS HIVDR assay is fit for clinical use, while allowing for enough flexibility in the accuracy, precision, and sequence error rates to allow for a variety of approaches to HIVDR testing. For example, we used Illumina-based sequencing in this study; however, some groups are developing platforms based on nanopore [42] and ion torrent [43] technologies that have higher sequencing error rates [44]. Although inherently more error-prone, these NGS platforms increase the accessibility to HIVDR testing with lower upfront costs and may allow for the adoption of NGS-based HIVDR assays in developing nations. Additionally, added quality control steps during data processing or the refinement of the technology may mitigate some of the current accuracy issues observed with these platforms. 

When we used our recommended assessment criteria and benchmarks to assess our HIVDR pipelines, we performed well overall; however, we fell short of our benchmark for accuracy when measuring DRMs at a frequency of 2%. This was likely due to the systematic error that was observed in our study that caused a general overestimation of DRM frequencies, which was present with all plasmid mixtures tested and across all runs. The reference materials used as input provided the most likely explanation for this bias. Because our tests analyzed plasmid mixtures, the accuracy of our HIVDR assays was limited by the accuracy of the qubit quantification and subsequent pooling of plasmids. In future experiments, we recommend that test panels are created using plasmid mixtures that have been quantified and further verified with qPCR [45] in combination with the qubit measurement. This highlights the need for high-quality reference materials for HIVDR assay validation and assessment. Although it was disappointing that our assay did not meet our goals for accuracy at the lower range of the measuring interval, we also believe this is encouraging as our assessment platform successfully identified a potential issue that could affect the clinical reporting of minority resistance variants. Because of this, we can work to discover the source of this bias to optimize our HIVDR assays for clinical use.

An added advantage of NGS for HIV genotyping is the ability to detect quasispecies that have multiple mutations, which in combination, affect drug resistance phenotypes, protein folding models, or subtype identification. In comparison, Sanger sequencing can only determine mutation frequencies at the population level. Template switching during reverse transcription and PCR introduces artifact recombinants that can make determining true recombinants versus artificial recombinants difficult, especially when observed at low frequencies. Therefore, extra precautions need to be taken when reporting DRMs that have combinatorial, synergistic, or antagonistic effects on the phenotype, especially when located distally across the HIV genome. Template switching can be minimized by altering the reverse transcription and PCR chemistry [46], through targeted RNase H mutations [47], or by decreasing the number of PCR cycles. Some recombination artifacts could be removed computationally [48,49], but this functionality is not currently an available feature of any HIVDR reporting tool. If shown to be clinically relevant, future validation criteria could assess the rate of template switching observed in individual HIVDR assays. Template switching could be exceedingly important in cases of high HIV diversity.

We also acknowledge several limitations in this study. Due to the lack of well-characterized wet panels in the field, our experiments on accuracy and sensitivity were conducted using only plasmid mixtures, which compared to clinical samples, avoided the error-prone RT step and required fewer cycles of PCR that would introduce sequence errors or PCR bias [14,50,51]. Because of this, it is likely that the accuracy and sensitivity of our HIVDR assays presented here are not reflective of what could be expected in a clinical setting. Meanwhile, there could be slight differences between the sensitivity readouts when it is measured from samples with all 34 mutations on the same molecule (as in this study) or from 34 molecules with each carrying one mutation (more likely seen in authentic clinical specimens) since the latter may be more affected by PCR errors or biases due to its higher “intra-host” diversity. As a solution, when possible, future studies could use well-characterized panel specimens that better approximate authentic clinical samples, as is standard practice with Sanger-based HIVDR proficiency test panels [8]. 

Viral RNA from infectious clones is also often used as reference materials to assess Sanger-based HIVDR assays and was included in our 70-specimen test panel. In this study, we used viral RNA from infectious clones as a way to measure the specificity of our NGS-based HIVDR assays. We identified multiple DRMs at frequencies below 5% that may have been introduced during viral propagation, which suggests infectious clones may not be a suitable reference material for sensitive NGS-based HIVDR assays. Alternatively, in vitro transcription of viral RNA, targeting the Pol region, and spike-ins into an appropriate matrix (plasma, serum, blood) would form high-quality reference materials for quality assessment and has been accomplished with partial or full genomes for other systems [51,52,53,54]. In-vitro-generated viral RNA would provide an excellent source of reference materials that can be used to better define the accuracy and specificity of HIVDR assays.

Additionally, future studies should investigate the effect of the input plasmid concentration and/or viral load on the criteria presented in this article, especially at the minimum input of 1000 copies/mL. With a smaller input concentration, sampling and PCR biases would have a significant additional impact on accuracy, precision, and sensitivity as the limit of detection for DRM abundance is ultimately determined by the input. This is a problem for any existing molecular test, including existing Sanger-based methods. For example, at viral loads of 1000 copies/mL, only 40 copies of HIV RNA are input into existing HIVDR protocols for low-volume dried blood spots. Based on this, our assay could only detect minority resistance variants at frequencies ≥2.5%, and an initial viral load of 2500 copies/mL would be required to detect mutations of ≥1% with limited accuracy. Therefore, extra precaution is needed when interpreting HIVDR mutations from samples with low viral loads, such as from patients on antiretrovirals. Logically, the assessment criteria and benchmarks will need to be adjusted for low-viral-load inputs. 

Even minority HIVDR variants at a prevalence of <0.5% may be associated with an increased risk of treatment failure [55]; however, the clinical significance of variants below 5% is highly debated and is summarized well in a systematic review by Mbunkah et al. [56]. In the future, if clinically relevant, it may be possible to detect variants at 0.5% or lower with high accuracy, especially with the use of unique molecular identifiers (UMIs) to identify PCR and sequencing errors and to correct for amplification biases [57,58]. As UMIs become more widely adopted and if sequence error rates continue to improve, the specific benchmarks suggested here may need to be revisited. This is especially true if further evidence builds that ultra-low-frequency variants have a significant impact on the response to treatment. 

In summary, a validated NGS-based HIVDR platform should meet the above-recommended benchmarks for linear range, precision, accuracy, sensitivity, and specificity. It should meet these requirements regardless of the major subtype and across the DRM measuring interval; however, some flexibility should be allotted when working with low-viral-load inputs (above 1000 copies/mL) when the test is constrained by sampling bias. Future work should investigate the effects of low viral load and poor sample quality on these performance characteristics.

## 5. Conclusions

As NGS is trending to become the dominant technology for HIVDR testing, there is a need for quality assessment criteria and benchmarks for assay validation. A well-established validation system will help to routinize HIVDR assays for resistance monitoring in clinical settings with a level of sensitivity that far exceeds the Sanger-based techniques currently in place. As a community, we need to move this technology forward as minority drug resistance variants overlooked by current methods may have a real impact on patient treatment efficacy. Here, we proposed an assessment platform that can be used to validate NGS-based HIVDR tests for clinical use and used it to evaluate our assays as a proof-of-concept. Similarly, we believe our proposed platform can be effectively applied to validate other quantitative HIVDR assays for clinical applications.

## Figures and Tables

**Table 1:** Coefficient of variation (CV) for intra-run, inter-run, inter-operator, and inter-lab precision.

	Coefficient of Variation (CV, %)			
% DRMs	Intra-Run	Inter-Run	Inter-Operator	Inter-Lab
1	12.7	5.2	2.8	15.1
2	6.9	10.3	1.7	15.9
5	7.7	5.3	3.0	13.1
10	6.2	4.6	1.6	15.7
20	8.2	7.2	5.9	7.7
100	0.1	0.3	0.2	0.1

Note: Values were calculated for all plasmid mixtures, with DRM frequencies of 1%, 2%, 5%, 10%, 20%, and 100%. Intra-run and inter-lab CVs were generally higher than the other factors tested.

**Table 2:** Proposed assessment criteria for NGS-based HIVDR assays and the recommended benchmarks.

Performance Characteristic	Proposed Definition for NGS-Based HIVDR	Suggested Benchmark	References
DRM Measuring Interval	The range of DRMs that can be detected with an acceptable linearity, sensitivity, and precision.	2–100%	[8,9,15]
Linear Range	The percentile range of DRM frequencies wherein the linear correlation is maintained between expected and observed values.	2–100%	[9,15,18,27,39]
Precision	The extent to which repeated testing on identical samples renders comparable results with acceptable repeatability and reproducibility.	CV < 25% (DRMs < 50%) CV < 15% (DRMs ≥ 50%)	[9,15,28,40]
Accuracy	The extent to which the detected DRM frequency is in agreement with reference materials. The value is relative to the theoretical DRM frequency.	Error < 40%	[9,15,40]
Sequence Error Rate	The overall error in amino acid frequencies due to PCR, sequencing, and data processing steps.	<2%	[8,36,41]
Analytical Sensitivity	The probability that the assay detects a DRM within the measuring interval when it is present (measured as 1—false negative rate).	>99% (plasmid)	[8,9,13,15,24]
Analytical Specificity	The probability that the assay does not detect a DRM when it is absent (measured as 1—false positive rate).	>95%	[9,13,15]
Limit of the Viral Load	The lowest viral load level at which the test can still effectively detect DRMs from a sample.	1000 copies/mL	[8,15,36]
Robustness	The capability of the assay to meet the above criteria using clinical samples of any major HIV subtype(s).	Coverage of all major subtypes	[8,13,15,36]

## Supplementary Materials

The following are available online at https://www.mdpi.com/1999-4915/12/6/627/s1, Figure S1: Probit linear regression analysis to determine the DRM limit of detection, Figure S2: Measurement of precision using clinical samples, Figure S3: Assay error across all runs, Table S1: The composition of the 70-specimen test panel used for HIVDR assay assessment, Table S2: Probit analysis for determining the limit of detection for NGS-based HIVDR assays, Table S3: Example of detected DRMs from the HIVDR testing of infectious clones.
