# Supplementary material for: Development and Application of Performance Assessment Criteria for Next-Generation Sequencing-Based HIV Drug Resistance Assays
Source: Viruses. 2020 Jun 10;12(6):627. doi: 10.3390/v12060627 (PMC7354553; doi:10.3390/v12060627)
Supplement: Supplementary file 1 [file viruses-12-00627-s001.zip › TableS2-S3-revised.docx]

**Table S2: Probit analysis for determining the limit of detection for HIV DRMs.**

| **Input DRM Percentage** | **Number of Replicates** | **Positive Replicates** | **Percent Positive** | **Probits** | **Comments** |
| --- | --- | --- | --- | --- | --- |
| 0 | 510 | 0 | 0.0% | 0 | Observed False Positive Rate = 0.0%; Specificity = ~100% |
| 1 | 510 | 475 | 93.10% | 6.48 | Observed False Negative Rate = 6.9%; Sensitivity = 93.1% |
| 2 | 510 | 510 | 100% | 8.09 | Observed False Negative Rate = 0%; Sensitivity = ~100% |
| 5 | 510 | 510 | 100% | 8.09 | Observed False Negative Rate = 0%; Sensitivity = ~100% |
| 10 | 510 | 510 | 100% | 8.09 | Observed False Negative Rate = 0%; Sensitivity = ~100% |
| 20 | 510 | 510 | 100% | 8.09 | Observed False Negative Rate = 0%; Sensitivity = ~100% |
| 100 | 510 | 510 | 100% | 8.09 | Observed False Negative Rate = 0%; Sensitivity = ~100% |

Notes: A probit regression analysis was used to determine the 95% confidence interval for detection of DRMs. These data were also used to determine analytical sensitivity and specificity at each of the DRM frequencies investigated. The 510 replicates here correspond to the 5 individual runs performed, 34 DRMs per specimen tested in triplicates for all runs. Of the 35 failed replicates at a 1% frequency, there was no discernable pattern across DRMs and the majority were identified within replicates performed at the National HIV and Retrovirology Laboratories (28/35).

**Table S3:** Example of detected DRMs from HIVDR testing of infectious clones. Results are from infectious clone KC109812. Several DRMs are shared between replicates suggesting they were introduced during viral replication prior
to HIVDR testing.

| Operator 1 | | | | Operator 2 | | | | NLHG | |
| --- | --- | --- | --- | --- | --- | --- | --- | --- | --- |
| Run #1 | | Run #2 | | Run #1 | | Run #2 | | Run #1 | |
| I47V | 98.47% | G48V | 1.19% | G48V | 2.35% | G48V | 1.09% | I47V | 98.72% |
| L33F | 99.08% | I47V | 97.18% | I47V | 92.86% | I47V | 95.96% | L33F | 98.95% |
| L90M | 99.38% | L33F | 97.82% | L33F | 91.52% | L33F | 96.31% | L90M | 99.70% |
| V32I | 98.90% | L90M | 99.64% | L90M | 99.34% | L90M | 99.49% | V32I | 98.89% |
| V82A | 99.31% | M46I | 1.13% | M46I | 4.03% | M46I | 2.32% | V82A | 99.24% |
|  |  | V32I | 97.42% | V32I | 90.23% | V32I | 95.75% |  |  |
|  |  | V82A | 99.28% | V82A | 98.69% | V82A | 99.08% |  |  |
